# ^18^F-Fluorodeoxyglucose positron emission tomography may not visualize radiation pneumonitis

**DOI:** 10.1186/s13550-019-0571-0

**Published:** 2019-12-19

**Authors:** Meiying Guo, Liang Qi, Yun Zhang, Dongping Shang, Jinming Yu, Jinbo Yue

**Affiliations:** 1 School of Medicine, Shandong University, Jinan, 250012 China; 2 Department of Radiation Oncology, Shandong Cancer Hospital and Institute, Shandong First Medical University and Shandong Academy of Medical Sciences, No. 440, Ji Yan Road, Jinan, 250117 China; 3 Equipment and material Department, Shandong Cancer Hospital and Institute, Shandong First Medical University and Shandong Academy of Medical Sciences, Jinan, 250117 China

**Keywords:** Radiation pneumonitis, Fluorodeoxyglucose, Positron emission tomography, Rat

## Abstract

**Background:**

Radiation pneumonitis is a common and potentially fatal complication of radiotherapy (RT). Some patients with radiation pneumonitis show increases in uptake of fluorodeoxyglucose (FDG) on positron emission tomography (PET), but others do not. The exact relationship between radiation pneumonitis and ^18^F-FDG PET findings remains controversial.

**Methods:**

We used an animal model of radiation pneumonitis involving both radiation and simulated bacterial infection in Wistar rats. Treatment groups (10 rats/group) were as follows: control, RT-only, lipopolysaccharide (LPS)-only, and RT+LPS. All rats had micro-PET scans at 7 weeks after RT (or sham). Histologic, immunohistochemical, and biochemical analyses were performed to evaluate potential mechanisms.

**Results:**

Irradiated rats had developed radiation pneumonitis at 7 weeks after RT based on pathology and CT scans. Maximum and mean standardized uptake values (SUV_max_ and SUV_mean_) at that time were significantly increased in the LPS group (*P* < 0.001 for both) and the RT+LPS group (*P* < 0.001 for both) relative to control, but were not different in the RT-only group (*P* = 0.156 SUV_max_ and *P* = 0.304 SUV_mean_). The combination of RT and LPS increased the expression of the aerobic glycolysis enzyme PKM2 (*P* < 0.001) and the glucose transporter GLUT1 (*P* = 0.004) in lung tissues. LPS alone increased the expression of PKM2 (*P* = 0.018), but RT alone did not affect PKM2 (*P* = 0.270) or GLUT1 (*P* = 0.989).

**Conclusions:**

Aseptic radiation pneumonitis could not be accurately assessed by ^18^F-FDG PET, but was visualized after simulated bacterial infection via LPS. The underlying mechanism of the model of bacterial infection causing increased FDG uptake may be the Warburg effect.

## Background

Radiation pneumonitis is a common and clinically significant side effect of definitive thoracic radiotherapy (RT) [1]. Severe radiation pneumonitis has a mortality rate approaching 50% [2]. Diagnosis and management of radiation pneumonitis after RT are complicated because of the lack of accepted, standardized criteria for diagnosis and because many patients have preexisting lung or cardiac disease [3]. An imaging method that accurately detects radiation pneumonitis would be quite valuable, but to date no reliable, reproducible method for doing so has been established. Computed tomography (CT) is widely used in current clinical practice but it is relatively insensitive for detecting early tissue injury and inflammation in the lung parenchyma.

The advent of molecular and functional imaging and the relationship between these imaging modalities and radiation pneumonitis have been the subject of considerable research interest. One such approach involves ^18^F- fluorodeoxyglucose (FDG) positron emission tomography (PET), a nuclear medicine medical imaging technique in wide use for diagnosis and disease staging and response evaluation in a great number of cancers as well as in several inflammatory conditions [4–6]. PET may be more useful than conventional imaging modalities because changes in tissue metabolism generally precede anatomic changes [7]. FDG PET imaging is being used for RT treatment planning, risk stratification, and response monitoring in locally advanced non-small-cell lung cancer [8, 9].

FDG PET can also accurately and sensitively detect inflammatory lesions, which is valuable for the diagnosis of rheumatoid arthritis, vasculitis, chondritis, lymphadenitis, ulcerative colitis, and other inflammatory conditions [10]. Inflammation due to bacterial infection is often mimicked in animal models by using lipopolysaccharide (LPS), which induces inflammatory cells to undergo a metabolic shift toward aerobic glycolysis, which resembles the Warburg effect first observed in cancer cells [11, 12]. Changes associated with the Warburg effect include increased glucose uptake and elevated glycolysis rate [13, 14], which form the basis of FDG PET scanning: imaging FDG relies on glucose uptake, and thus some infections and inflammatory processes can be accurately visualized by FDG-PET. However, the essence of radiation pneumonitis is aseptic inflammation induced by radiation-activated inflammatory factors, which seems to result from a different mechanism than bacterial inflammation. FDG uptake has been linked with radiation dose [15]. Abdulla and colleagues showed that the mean standard uptake value (SUV_mean_) in lung parenchyma on FDG PET may be useful for quantifying lung inflammation after thoracic RT [16]. However, to date, no evidence is available to support the use of PET for the diagnosis of radiation pneumonitis. In the clinic, some patients with radiation pneumonitis manifest increases in FDG uptake, but others do not. The exact relationship between radiation pneumonitis and findings on FDG PET remains controversial.

Thus, we undertook this study to (1) investigate the role of FDG PET for characterizing radiation-induced lung injury based on pathologic findings under well-controlled experimental conditions and (2) explore potential mechanisms underlying changes in FDG uptake caused by radiation-induced and simulated bacterial pneumonitis in an animal model.

## Methods

We used a rat model of radiation-induced and simulated bacterial lung inflammation to study the relationship between FDG PET and pneumonitis under controlled experimental conditions. Eight-week-old male Wistar rats (purchased from the Experimental Animal Center of Shandong University) were housed under specific pathogen-free conditions with free access to food and water. All animal experiments were done according to a protocol approved by the Committee for the Care and Use of Laboratory Animals of Shandong University and complied with the ARRIVE guidelines.

### Treatments

To confirm the feasibility of the proposed experiments, 14 male Wistar rats underwent CT-based treatment simulation, treatment planning, and treatment delivery, followed by weekly CT scanning and pathologic evaluation of lung tissues to confirm the presence of pneumonitis. RT simulation was done while the rats were restrained in the prone position in a specialized device, with 3 markers used to determine the radiation target (Additional file 1: Fig. S1). CT images were obtained with a Philips Brilliance Big Bore system with bellows (Philips Healthcare, DA Best, The Netherlands) and transmitted to a Varian Eclipse 10.0 Treatment Planning System. Every rat had an independent RT plan, for which a radiation oncologist contoured the entire right lung, and a physicist developed the plan. RT was delivered with 6-MV X-rays, to a total dose of 20 Gy in a single fraction, with a Varian Unique Medical Linear Accelerator while the rats were anesthetized with 2% pentobarbital sodium at a dose of 30 mg/kg (Sigma-Aldrich Co. LLC, St. Louis, MO, USA). In fact, this dose was selected according to the previous published studies, in which they declared that a single dose of 20 Gy develops considerable radiation lung injury [17, 18]. The left lung was shielded to minimize radiation exposure. Target regions were irradiated with a margin of at least 2 mm from the center to prevent radiation esophagitis or myelitis. Other regions were protected from irradiation with a multileaf collimator. CT scans were obtained weekly to monitor the progress of radiation pneumonitis, and at 1 day, 1 week, 2 weeks, 4 weeks, 6 weeks, and 7 weeks after the RT, two rats per treatment group (see below) were killed and their lungs examined for pathological evidence of radiation-induced lung injury. We determined the 7-week study period through weekly CT scanning and pathologic evaluation. Under our experimental conditions, almost all the rats could develop radiation pneumonitis at this time point, which was also consistent with the 6–8 weeks study period reported in the previously published literature [17–20]. Radiation pneumonitis refers to interstitial pneumonitis that occurs after radiotherapy, not caused by other factors such as bacterial infection, usually manifested as diffuse alveolar wall thickening and the accumulation of inflammatory cells.

In the formal experiment, 40 male Wistar rats weighing 178–220 g each were randomly assigned to one of four treatment groups (10 rats/group): control, RT-only, LPS-only, and RT plus LPS. RT was planned and delivered as described above. LPS (obtained from Solarbio Life Sciences, Beijing, China) was given as a single intranasal injection of 5 mg/kg body weight [10] at 24 h before the micro-PET scan. All rats had micro-PET scans at 7 weeks after RT (or sham). All experiments were repeated twice, and all findings were similar across all experiments.

### Micro-PET imaging

^18^F-FDG was synthesized according to the method developed by Ido [21] to a purity of > 95% in the Jiangsu Atomic Energy Laboratory (Jiangsu, China). Micro-PET images were acquired with an Inveon PET scanner (Siemens Preclinical Solutions, LLC, Knoxville, TN, USA). Before scanning, rats were fasted for 8–12 h, after which 7.4–11.1 MBq of ^18^F-FDG was injected via the tail vein and PET scans were obtained 60 min later. Anesthesia was maintained during the PET scans with 1.5% isoflurane in 100% oxygen at 1.5 L/min. For the scans, rats were placed prone on the bed of micro-PET, and the limbs were fixed with tape. A 10-min static single-frame scan was acquired with a small-animal PET camera, and images were reconstructed by ordered subsets expectation maximization (OSEM)-3D IAW (Siemens Preclinical Solutions).

Two experienced nuclear medicine physicians examined all PET images in a double-blinded fashion. Regions of interest (ROIs) in the right lungs were drawn using vendor software (IS_v1.4.3 SP1; Siemens Healthineers, Erlangen, Germany). The SUV was calculated as an absolute measure of ^18^F-FDG uptake in an ROI as:

[(measured activity concentration, in kBq/mL)/(injected dose, in kBq/body weight, in g)].

All animals were weighed before scanning. SUV_max_ and SUV_mean_ were defined as the maximum and mean tracer uptake in the ROIs.

### Pathologic evaluations

After the micro-PET scan, rats were killed by anesthetizing, and the right lungs were removed, fixed overnight in 10% formalin, and embedded in paraffin. Serial 4-μm tissue sections were prepared for histologic analysis and immunohistochemical staining of PKM2 and GLUT1, detailed information was provided in the Additional file 1. Digital images of the immunohistochemical-stained slides were acquired with a Nikon Eclipse 80i microscope equipped with Nikon DS-Fi1 camera. All images were taken under high magnification (× 400) with consistent imaging parameters (uniform light source, exposure time, and autofocus). Expression levels of PKM2 and GLUT1 were measured with Image-Pro Plus (version 6.0, Media Cybernetics, Rockville, MD, USA) and quantified as integrated optical density (IOD)/area.

Concentrations of interleukin (IL)-1, IL-6, and transforming growth factor (TGF)-β in serum and bronchoalveolar lavage fluid (BALF) from the right (irradiated) and left (unirradiated) lungs were measured with a Rat IL-1 ELISA Kit, a Rat IL-6 ELISA Kit, and a Rat TGF-β ELISA Kit (Abcam, Cambridge, MA, USA) according to the manufacturer’s instructions. Plates were read with a VersaMaxMultiplate spectrophotometric reader at 450 nm.

Lactate and pyruvate levels were measured in blood samples with assay kits provided by Nanjing Jiancheng Bioengineering Institute (Nanjing, China) according to protocol instructions. Concentrations of lactate and pyruvate were evaluated by measuring the absorbance (*A*) at 530 nm and 505 nm and calculated as:

Lactate content (mmol/L) = [*A*_530_(sample) – *A*_530_(blank)]/[*A*_530_(standard) – *A*_530_(blank)] × standard concentration (3 mmol/L) × sample dilution multiple;

Pyruvate content (*μ*mol/mL) = [*A*_505_(sample) – *A*_505_(blank)]/[*A*_505_(standard) – *A*_505_(blank)] × standard concentration (0.2 *μ*mol/mL) × sample dilution multiple.

### Statistical analysis

Statistical analyses were done with SPSS 23.0 (SPSS Inc., Chicago, IL, USA). Differences between two groups were assessed with two-tailed paired *t* tests. One-way analysis of variance and Bonferroni’s post hoc test were used for multiple comparisons. Pearson correlation analysis was used to evaluate associations between groups. The threshold for statistical significance was set at *P* < 0.05.

## Results

### Pneumonitis and cytokine levels

**Fig. 1:**
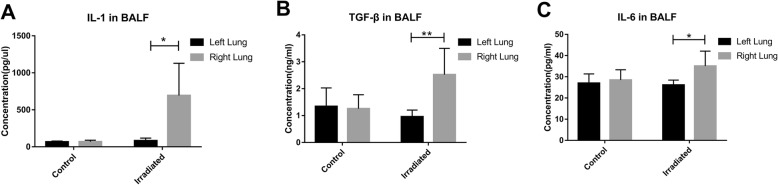
Concentrations of inflammatory cytokines in bronchoalveolar lavage fluid (BALF) samples from the left and right lungs of control rats and irradiated rats. **a** Interleukin (IL)-1. **b** transforming growth factor-beta (TGF-β). **c** IL-6. *P < 0.05, **P < 0.01

In the pre-experiment to determine the rat model of radiation pneumonitis, significant radiation pneumonitis was apparent on weekly micro-CT scans by 7 weeks after RT (Additional file 1: Fig. S2). Pathologic changes during this period are shown in Additional file 1: Fig. S3. At 7 weeks after RT, plasma levels of IL-1α and TGF-β in irradiated rats were significantly higher than in non-irradiated rats (*P* = 0.006 and *P* = 0.006), but receipt of RT did not affect plasma IL-6 levels (*P* = 0.951) (Additional file 1: Fig. S4). In irradiated rats, BALF samples from the right (irradiated) lung had higher levels of IL-1α, IL-6, and TGF-β than in BALF from the left (non-irradiated) lung (*P* = 0.014, *P* = 0.027, and *P* = 0.008, respectively). However, in non-irradiated rats, no differences were found in IL-1α, IL-6, or TGF-β in BALF from the left and right lungs (*P* = 0.819, *P* = 0.624, and *P* = 0.832, respectively) (Fig. 1).

### Micro-PET findings

**Fig. 2:**
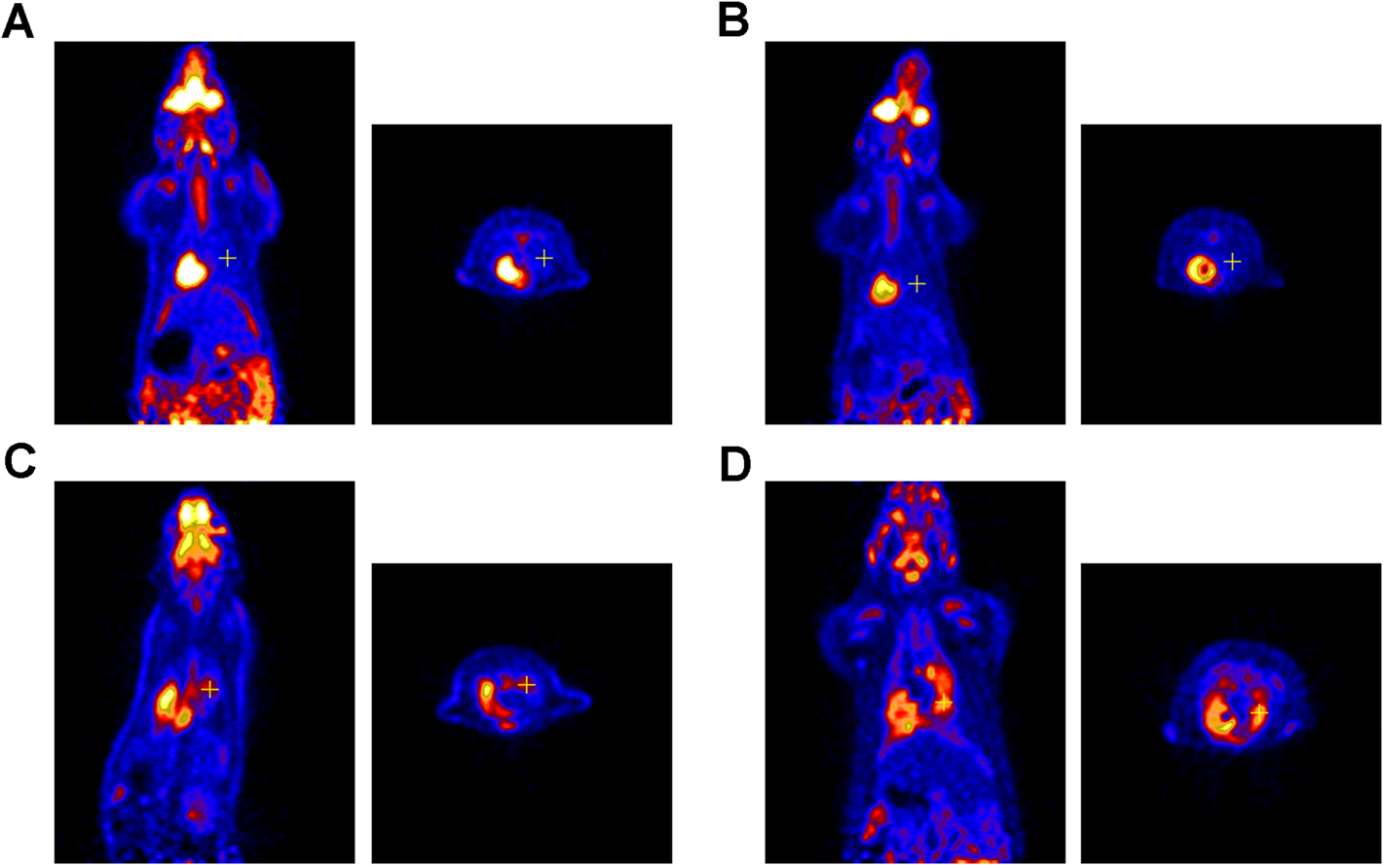
Coronal (left) and cross-sectional scans of FDG uptake in the right lungs of rats in the control, radiotherapy (RT)-only, lipopolysaccharide (LPS)-only, and RT+LPS groups at 7 weeks after RT. No significant FDG uptake was observed in the control (**a**) or RT-only group (**b**), but FDG uptake clearly increased in the LPS-only (**c**) and RT+LPS (**d**) groups

**Fig. 3:**
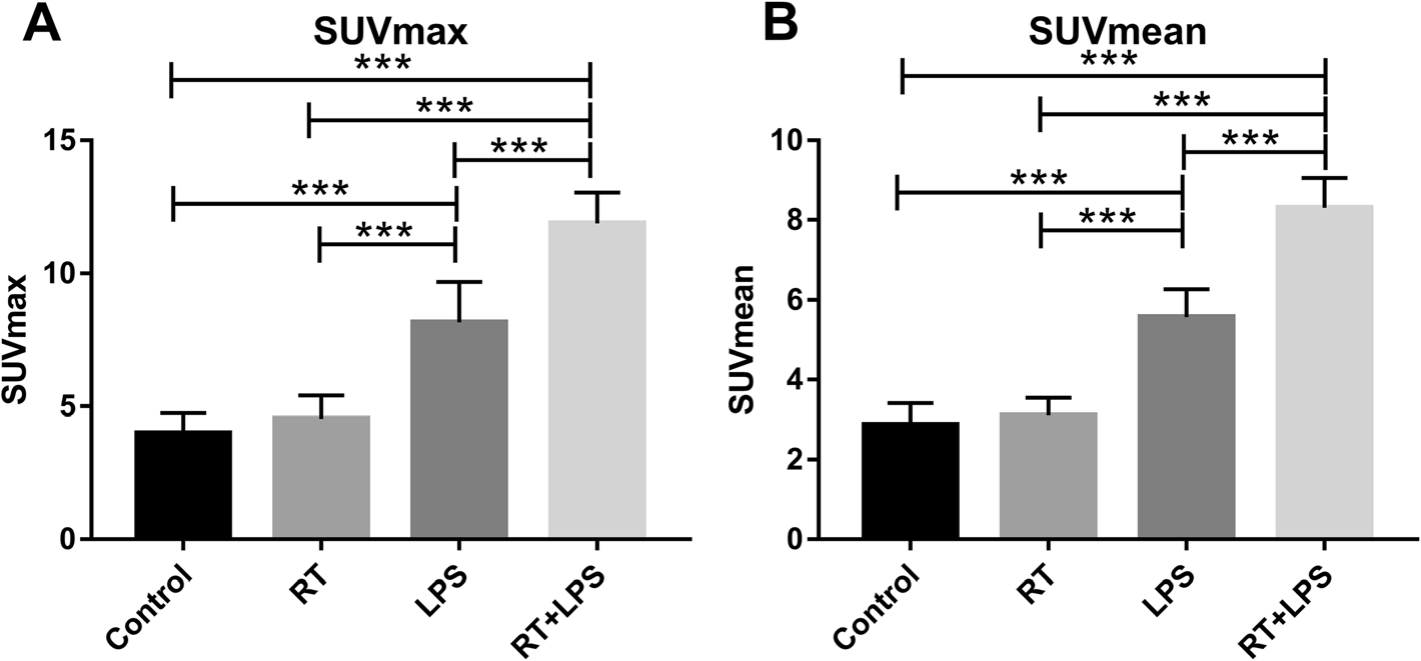
Maximum and mean standardized uptake values (SUV_max_ and SUV_mean_) in the right lung in the four treatment groups. **a** SUV_max_ in the control, radiotherapy (RT)-only, lipopolysaccharide (LPS)-only, and RT+LPS groups. **b** SUV_mean_ in the control, RT-only, LPS-only, and RT+LPS groups. Data represent means ± SEM, for 10 rats per group. ***P < 0.001

Micro-PET scans were obtained from all 40 rats (10 per group). No significant FDG uptake was observed in the RT-only and control groups, but the RT+LPS and LPS-only groups had obvious FDG uptake in the right lung (Fig. 2). The SUV_max_ value was highest in the RT+LPS group (11.87 ± 1.16) and was significantly higher than that in the LPS group (8.16 ± 1.53, *P* < 0.001), the RT-only group (4.53 ± 0.87, *P* < 0.001), and the control group (3.99 ± 0.75, *P* < 0.001). SUV_max_ in the LPS-only group was also higher than that in the RT-only and control groups (*P* < 0.001 for both), but was not different in the RT-only and control groups (*P* = 0.156) (Fig. 3a). Similar results were also found for SUV_mean_, with values of 2.87 ± 0.55 (control), 3.10 ± 0.44 (RT-only), 5.57 ± 0.70 (LPS-only), and 8.30 ± 0.75 (RT+LPS). Compared with the control group, SUV_mean_ was significantly elevated in the RT+LPS and LPS-only groups (*P* < 0.001 for both), but were not different in the RT-only group (*P* = 0.304) (Fig. 3b).

### Proteins in the aerobic glycolysis pathway

**Fig. 4:**
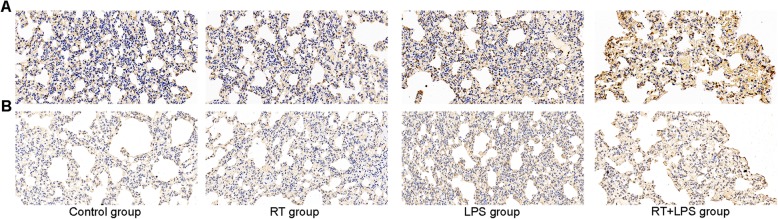
Immunohistochemical stains for PKM2 (**a**) and GLUT1 (**b**) in tissues from the right lungs of rats in the control, radiotherapy (RT)-only, lipopolysaccharide (LPS)-only, and RT+LPS groups (×400)

**Fig. 5:**
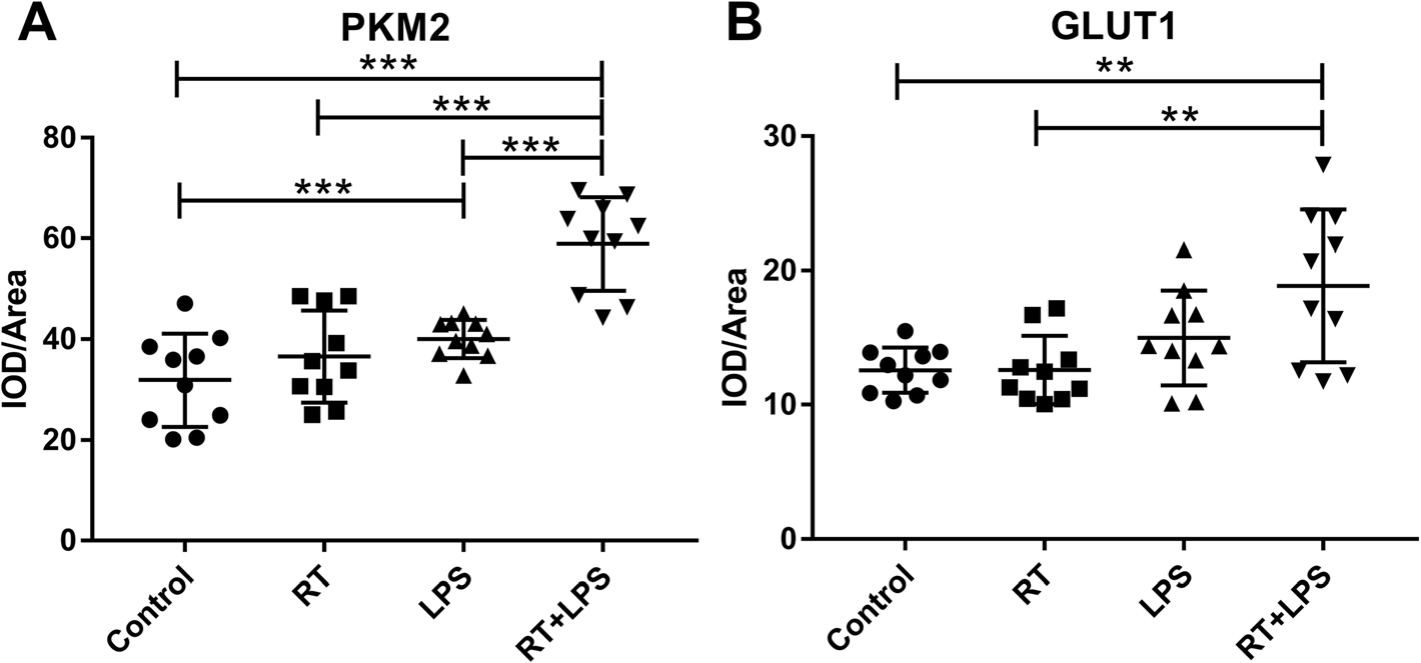
Integral optical density (IOD)/area of PKM2 (**a**) and GLUT1 (**b**) in the four treatment groups: control, radiotherapy (RT)-only, lipopolysaccharide (LPS)-only, and RT+LPS groups. **P < 0.01, ***P < 0.001

To explore mechanisms that could affect FDG uptake, we investigated two important proteins in the aerobic glycolysis pathway, PKM2 and GLUT1 (Fig. 4). PKM2 is the main rate-limiting enzyme in glycolysis, and its methylation acts to switch from oxidative phosphorylation to aerobic glycolysis. In the current study, PKM2 was increased in the LPS-only and RT+LPS groups compared with control (*P* = 0.018 and *P* < 0.001), but RT-only did not affect PKM2 expression (*P* = 0.270) (Fig. 5a). GLUT1 is a transporter on the surface of cell membranes that can transport glucose directly into cells. Compared with control, GLUT1 expression was higher in the RT+LPS group (*P* = 0.004) but was not different in the LPS-only (*P* = 0.068) or RT-only (*P* = 0.989) groups (Fig. 5b).

**Fig. 6:**
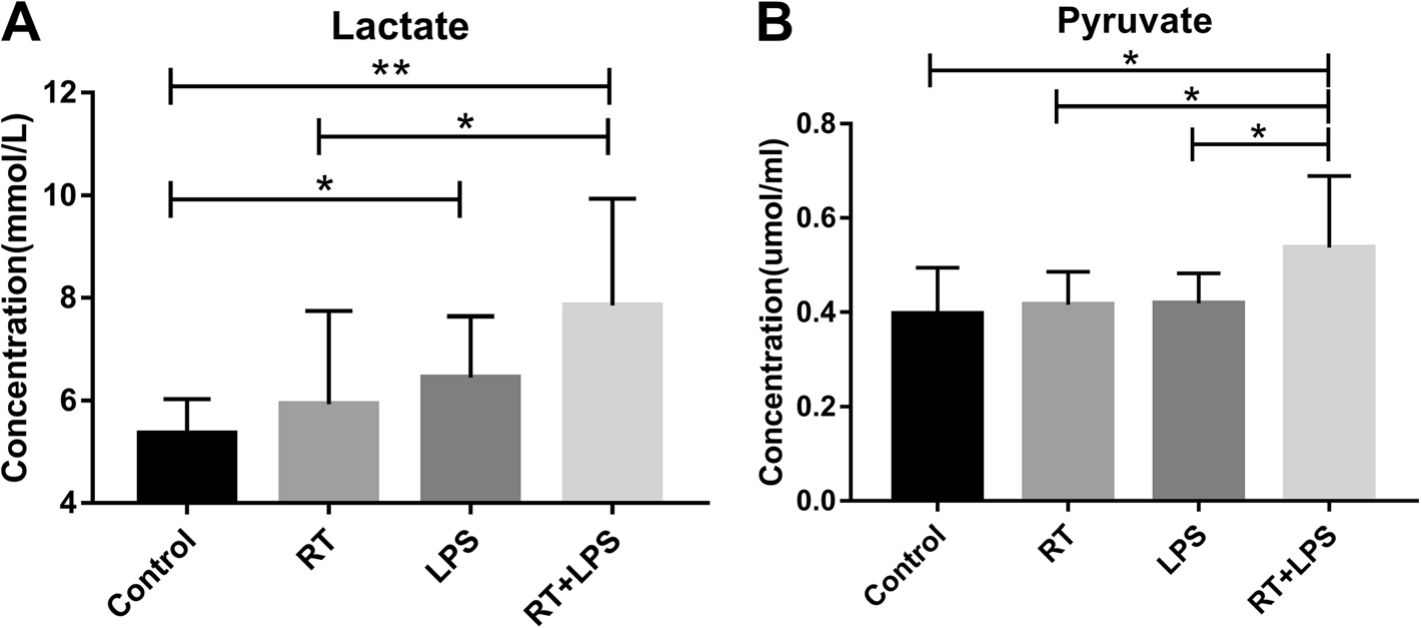
Plasma concentrations of lactate (**a**) and pyruvate (**b**) in the four treatment groups: control, radiotherapy (RT)-only, lipopolysaccharide (LPS)-only, and RT+LPS groups. *P < 0.05, **P < 0.01

We also assessed levels of lactate, the end product of aerobic glycolysis, in serum samples. Mean serum lactate levels were 5.36 ± 0.21 mmol/L in the control, 5.93 ± 0.57 mmol/L in the RT-only, 6.45 ± 0.38 mmol/L in the LPS-only, and 7.85 ± 0.66 mmol/L in the RT+LPS groups; serum lactate levels were higher in the RT+LPS group than in the control (*P* = 0.002) or RT-only (*P* = 0.021) groups but were not different from those in the LPS-only group (*P* = 0.084) (Fig. 6a). We also measured serum levels of pyruvate, an intermediate product of glucose metabolism that can be used to complete the tricarboxylic acid cycle or aerobic glycolysis, and found that serum pyruvate levels did not change after RT-only or LPS-only (*P* = 0.617 or *P* = 0.562) but were higher in the RT+LPS group (*P* = 0.024 vs. control) (Fig. 6b).

### Correlation analysis of FDG uptake, PKM2, GLUT1, lactate, and pyruvate levels

**Fig. 7:**
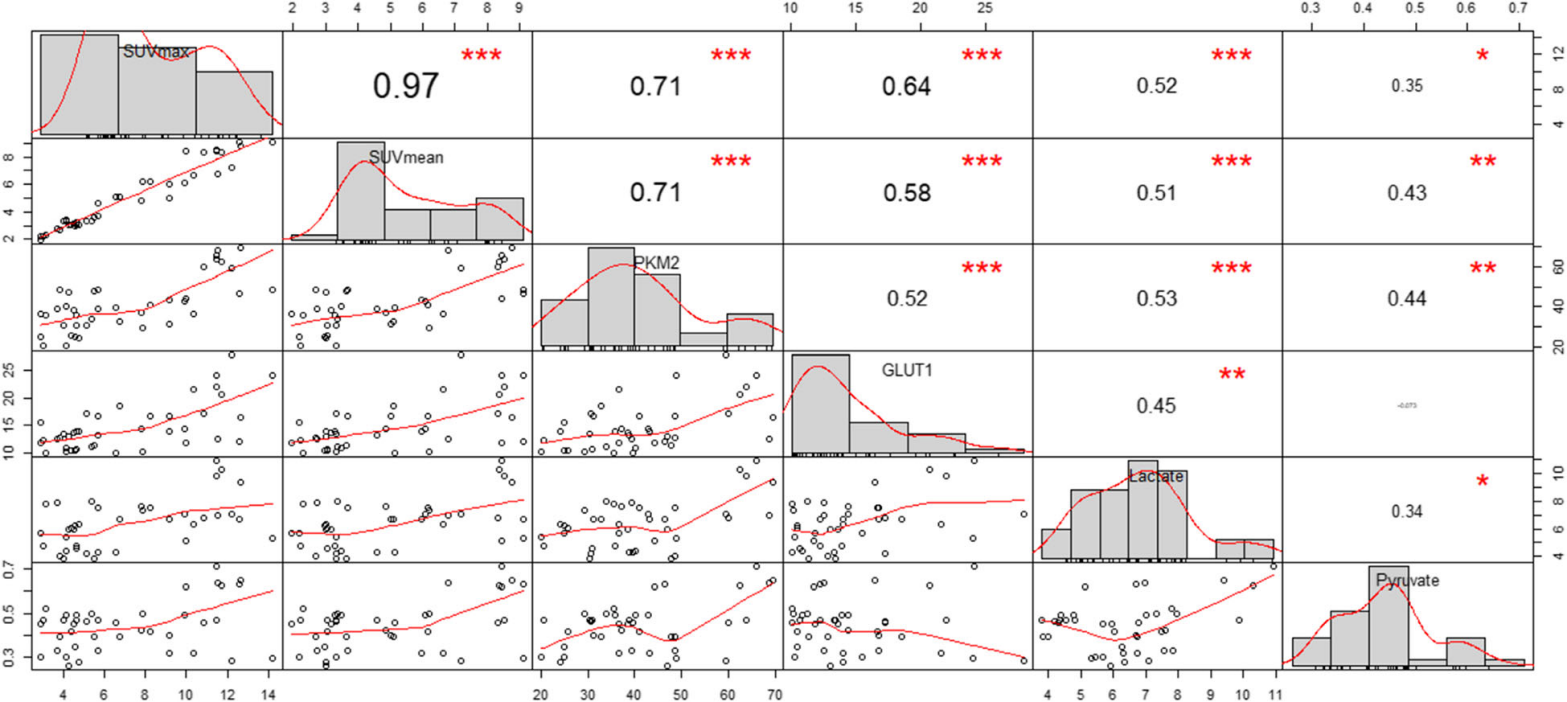
The diagonal line represents the distribution of each variable, with the correlation coefficient above the diagonal and the correlation curve below the diagonal. *P < 0.05, **P < 0.01, and ***P < 0.001

Pearson correlation analysis indicated that SUV_max_ was strongly related to PKM2, GLUT1, and lactate (*r* = 0.71 *P* < 0.001 [PKM2], *r* = 0.64 *P <* 0.001 [GLUT1], and *r* = 0.52 *P* < 0.001 [lactate]) (Fig. 7). Similar results were found for SUV_mean_ (*r* = 0.71 *P* < 0.001 [PKM2], *r* = 0.58 *P* < 0.001 [GLUT1], and *r* = 0.51 *P* < 0.001 [lactate]). PKM2, the main rate-limiting enzyme in glycolysis, correlated with GLUT1 (*r* = 0.52, *P* < 0.001) and lactate (*r* = 0.53, *P* < 0.001). Pyruvate, an intermediate in aerobic glycolysis, was not correlated with other factors, especially GLUT1 (*r* = − 0.073, *P* = 0.814). In sum, both ^18^F-FDG uptake measurements (SUV_max_ and SUV_mean_) correlated strongly with immunohistochemical markers and lactate levels, suggesting that the Warburg effect had been triggered in response to LPS, but not radiation.

## Discussion

In this comprehensive evaluation of ^18^F-FDG PET for assessing radiation-induced lung injury, micro-PET scans showed no increases in FDG uptake after RT-only versus control, suggesting that radiation pneumonitis itself, which is essentially interstitial aseptic pneumonia, cannot be accurately assessed by FDG-PET. However, we did find elevated FDG uptake in rats given LPS, with or without RT, which we interpret as indicating that the model of bacterial infection simulated by LPS affects the normal glucose metabolism and uptake process via the Warburg effect, leading to upregulation of the expression of the GLUT1 glucose transporter on the cell membrane, which led to increased ^18^F-FDG uptake. Based on above findings, our study suggested that radiation pneumonitis with elevated FDG may be associated with bacterial infections, requiring further detection of pathogens and strengthened treatment.

Radiation-induced lung injury is a well-known comorbidity of thoracic RT for the lung, esophageal, breast, thymic, and hematologic cancer [22]. Early and accurate diagnosis of radiation pneumonitis is particularly important. At present, the diagnosis of radiation pneumonitis is based mainly on symptoms, history and time since RT, and occasionally imaging findings. Chest X-ray and CT are the most commonly used, but both imaging methods have poor specificity and sensitivity for radiation pneumonitis [3, 23]. Several groups have investigated FDG-PET for assessing radiation-induced lung inflammation [16, 24–27]. However, to date, no study has clearly indicated whether ^18^F-FDG PET is useful for the diagnosis of radiation pneumonitis. To the best of our knowledge, this study was the first to explore the role of ^18^F-FDG PET for diagnosing radiation-induced lung injury, with pathological examinations used as the gold standard and excluding confounding factors in an animal model.

In our feasibility study, SUV_max_ and SUV_mean_ in the right lung did not increase after RT, although histologic evaluation confirmed the presence of pathological changes associated with radiation pneumonitis in the irradiated rats, and obvious pneumonia lesions could also be observed on CT. Because all of the rats were maintained in a sterile environment and confounding factors such as bacterial or fungal infection were excluded, we considered that these lesions represented aseptic interstitial radiation pneumonitis and concluded that ^18^F-FDG PET may not be able to visualize aseptic radiation pneumonitis.

Radiation pneumonitis is a complex process; its essence is aseptic inflammation. Rubin and Casarett first described the classical mechanism of radiation pneumonitis, which was later detailed by others [28–31]. Radiation pneumonitis always occurs in radiation fields, possibly due to the direct cytotoxic action of ionizing radiation on lung cells. Immediately after radiation exposure, increased capillary permeability contributes to pulmonary edema. Damage to type I and II pneumocytes results in loss of surfactant and infiltration of serum proteins into the alveoli. Cytokines released from damaged lung cells (such as IL-1, IL-6, or TNF-α) attract inflammatory cells to the alveoli and pulmonary interstitium, inducing the acute phase pneumonitis [32]. However, our results lead us to speculate that the interstitial pneumonia experienced after a radiation-induced cytotoxic action on lung cells does not cause changes in glucose metabolism, and thus cannot be visualized by FDG-PET.

We also found that the SUV_max_ and SUV_mean_ in the right lungs of the rats were elevated in the LPS and RT+LPS groups. LPS is a major component of the outer membrane of Gram-negative bacteria and is the prototypical endotoxin responsible for the clinical manifestations of Gram-negative bacterial infections [33]. Our results suggest that the model of bacterial infection process simulated by LPS can cause changes in glucose metabolism resulting in changes in FDG uptake, a finding consistent with another report that ^18^F-FDG PET can accurately and sensitively detect inflammatory lesions [10]. However, the observation that SUV_max_ and SUV_mean_ values were higher in the RT+LPS group than in the LPS group suggests that radiation’s actions on lung parenchymal cells may exacerbate the manifestations of bacterial infection.

Several groups have investigated FDG-PET for assessing radiation-induced lung inflammation [16, 24–26]. Castillo R. et al. indicated that the pre-treatment pulmonary FDG uptake, as quantified by the SUV(95), could predict symptoms of radiation pneumonitis in both lung cancer and esophageal cancer [34, 35]. However, pre-treatment pulmonary FDG uptake mainly depends on the metabolic activity in the tumor and background, and cannot reflect the true relationship between FDG uptake and radiation pneumonitis. Besides, some clinical studies observed the positive correlation of post-treatment FDG uptake and radiation pneumonitis [36–38]. But in fact, the result showed that the true positive rate was only 0.67 [37]. Similarly, a study from Australia found that the intensity of FDG uptake in pulmonary tissue 70 days after RT showed significant correlation with the presence and severity of radiation pneumonitis [38]. But in this study, of the 21 patients who did not have increased FDG uptake, seven actually had radiation pneumonitis, and of the 53 patients without radiation pneumonitis, 39 showed an increase in FDG uptake. Above studies indicated that FDG uptake and radiation pneumonitis are not one-to-one correspondence. According to our findings, bacterial infections may be one of the main causes of confusion, as radiation pneumonitis is often associated with infection.

The Warburg effect refers to the observation that even under aerobic conditions, cancer and other proliferating cells tend to favor metabolism via glycolysis rather than the much more efficient oxidative phosphorylation [39], resulting in increases in glucose uptake and lactate production that can be visualized in malignant tumors on FDG PET scanning [40]. Because the inflammatory response can also prompt the Warburg effect [13], we deduced that the increased FDG uptake caused by LPS was driven by the Warburg effect of inflammatory cells. Consistent with this supposition, LPS+RT led to increases in PKM2 expression but RT alone did not, and GLUT1 expression followed the same trend. Moreover, the increased levels of lactate and pyruvate in the LPS+RT group but not in the RT-only group further support this concept [41], as do the results of our Pearson correlation analysis indicating correlations between PKM2/GLUT1 and SUV_max_/SUV_mean_. Collectively, these findings suggest that the Warburg effect may be the mechanism driving the inflammation from LPS, and not radiation pneumonitis, that led to the changes seen on ^18^F-FDG PET in our study.

A strength of our study was that we use RT simulation, planning, and delivery to induce targeted radiation pneumonitis, which resembles the clinical situation better than the previous use of ordinary X-ray irradiation to simulate RT, which is much less accurate [42]. Moreover, we obtained pathologic confirmation that all irradiated rats developed radiation pneumonitis, which we also verified by measuring changes in cytokines such as IL-1 and TGF-β in BALF samples. Our model system may prove useful for future tests of radiation pneumonitis by others.

Although we were able to determine that radiation pneumonitis could not be accurately assessed by FDG-PET, this study had several limitations. First, we did not examine human patients, only rats, and thus our findings on FDG uptake in lungs may not sufficiently reflect the human condition. Second, unlike the brain, heart, and liver, which are highly metabolically active, the lung has lower baseline FDG uptake. Further, the lack of change in FDG uptake by the irradiated lungs after RT made delineating ROIs on the post-treatment scans difficult, which may have introduced calculation errors. Finally, the number of animals used in this study was small, and our approach should be verified with larger numbers of animals.

## Conclusions

In this comprehensive evaluation of ^18^F-FDG PET for imaging radiation-induced lung injury, we found that aseptic radiation pneumonitis could not be accurately assessed by ^18^F-FDG-PET, but the model of radiation pneumonitis accompanied with bacterial infection simulated by LPS could be visualized in FDG-PET. The mechanism underlying these results may be the Warburg effect, which promotes conversion of glucose metabolism from the tricarboxylic acid cycle to aerobic glycolysis and leads to increases in glucose uptake. RT alone did not activate the Warburg effect but could exacerbate the pulmonary effects of bacterial infection.

## Supplementary information

**Additional file 1: **Supplementary Materials. **Fig. S1.** (A) When performing positioning and radiotherapy, experimental rats were positioned prone in special fixture, three markers were used to determine the location; (B) experimental rats undergoing radiotherapy. **Fig. S2.** CT scans of irradiated lungs from pre-radiation to 7 weeks after radiotherapy. **Fig. S3.** Histological changes of irradiated lungs from pre-radiation to 7 weeks after radiotherapy. **Fig. S4.** Concentrations of inflammatory factors including IL-1, IL-6 and TGF-β in plasma between control rats and irradiated rats. (A) Concentration of IL-1; (B) concentration of TGF-β; (C) concentration of IL-6. **P < 0.01.

## Data Availability

Data sharing is not applicable to this article as no datasets were generated or analyzed during the current study.

## Abbreviations

A: Absorbance
BALF: Bronchoalveolar lavage fluid
CT: Computed tomography
FDG: Fluorodeoxyglucose
IL: Interleukin
IOD: Integrated optical density
LPS: Lipopolysaccharide
OSEM: Ordered subsets expectation maximization
PET: Positron emission tomography
ROI: Region of interest
RT: Radiotherapy
SUV: Standardized uptake value
TGF: Transforming growth factor

## References

[1] 2nd SC. Thoracic radiation normal tissue injury. Semin Radiat Oncol. 2017; 27: 370. 10.1016/j.semradonc.2017.04.009 28865520

[2] Wang JY, Chen KY, Wang JT (2002). Outcome and prognostic factors for patients with non-small-cell lung cancer and severe radiation pneumonitis. Int J Radiat Oncol Biol Phys.

[3] Kocak Z, Evans ES, Zhou SM (2005). Challenges in defining radiation pneumonitis in patients with lung cancer. Int J Radiat Oncol Biol Phys.

[4] Van den Wyngaert T, Helsen N, Carp L (2017). Fluorodeoxyglucose-positron emission tomography/computed tomography after concurrent chemoradiotherapy in locally advanced head-and-neck squamous cell cancer: the ECLYPS study. J Clin Oncol.

[5] Cavo M, Terpos E, Nanni C (2017). Role of (18)F-FDG PET/CT in the diagnosis and management of multiple myeloma and other plasma cell disorders: a consensus statement by the International Myeloma Working Group. Lancet Oncol.

[6] Yamamura K, Izumi D, Kandimalla R (2019). Intratumoral fusobacterium nucleatum levels predict therapeutic response to neoadjuvant chemotherapy in esophageal squamous cell carcinoma. Clin Cancer Res.

[7] Gupta NC, Tamim WJ, Graeber GG (2001). Mediastinal lymph node sampling following positron emission tomography with fluorodeoxyglucose imaging in lung cancer staging. Chest.

[8] Salavati A, Duan F, Snyder BS (2017). Optimal FDG PET/CT volumetric parameters for risk stratification in patients with locally advanced non-small cell lung cancer: results from the ACRIN 6668/RTOG 0235 trial. Eur J Nucl Med Mol Imaging.

[9] Geiger GA, Kim MB, Xanthopoulos EP (2014). Stage migration in planning PET/CT scans in patients due to receive radiotherapy for non–small-cell lung cancer. Clin Lung Cancer.

[10] Kubota K, Yamashita H, Mimori A (2017). Clinical value of FDG-PET/CT for the evaluation of rheumatic diseases: rheumatoid arthritis, polymyalgia rheumatica, and relapsing polychondritis. Semin Nucl Med.

[11] Altenberg B, Greulich KO (2004). Genes of glycolysis are ubiquitously overexpressed in 24 cancer classes. Genomics.

[12] Raulien N, Friedrich K, Strobel S (2017). Fatty acid oxidation compensates for lipopolysaccharide-induced Warburg effect in glucose-deprived monocytes. Front Immunol.

[13] Palsson-McDermott EM, Curtis AM, Goel G (2015). Pyruvate kinase M2 regulates Hif-1alpha activity and IL-1beta induction and is a critical determinant of the Warburg effect in LPS-activated macrophages. Cell Metab.

[14] Tannahill GM, Curtis AM, Adamik J (2013). Succinate is an inflammatory signal that induces IL-1beta through HIF-1alpha. Nature.

[15] Shusharina N, Liao Z, Mohan R (2018). Differences in lung injury after IMRT or proton therapy assessed by ^18^FDG PET imaging. Radiother Oncol.

[16] Abdulla S, Salavati A, Saboury B (2014). Quantitative assessment of global lung inflammation following radiation therapy using FDG PET/CT: a pilot study. Eur J Nucl Med Mol Imaging.

[17] Zanette B, Stirrat E, Jelveh S (2018). Physiological gas exchange mapping of hyperpolarized (129) Xe using spiral-IDEAL and MOXE in a model of regional radiation-induced lung injury. Med Phys.

[18] Wang J, Zhou F, Li Z (2018). Pharmacological targeting of BET proteins attenuates radiation-induced lung fibrosis. Sci Rep.

[19] Medhora M, Haworth S, Liu Y (2016). Biomarkers for radiation pneumonitis using noninvasive molecular imaging. J Nucl Med.

[20] Doganay O, Stirrat E, McKenzie C (2016). Quantification of regional early stage gas exchange changes using hyperpolarized (129) Xe MRI in a rat model of radiation-induced lung injury. Med Phys.

[21] Ido T, Wan C-N, Casella V (1978). Labeled 2-deoxy-D-glucose analogs. 18F-labeled 2-deoxy-2-fluoro-D-glucose, 2-deoxy-2-fluoro-D-mannose and 14C-2-deoxy-2-fluoro-D-glucose. J Labelled Comp Radiopharm.

[22] Bledsoe TJ, Nath SK, Decker RH (2017). Radiation pneumonitis. Clin Chest Med.

[23] Eda Y, Higginson DS, Merdan F (2012). Challenges scoring radiation pneumonitis in patients irradiated for lung cancer. Lung Cancer.

[24] Echeverria AE, Mccurdy M, Castillo R (2013). Proton therapy radiation pneumonitis local dose–response in esophagus cancer patients. Radiother Oncol.

[25] Hart JP, Mccurdy MR, Muthuveni E (2008). Radiation pneumonitis: correlation of toxicity with pulmonary metabolic radiation response. Int J Radiat Oncol Biol Phys.

[26] Guerrero T, Johnson V, Hart J (2007). Radiation pneumonitis: local dose versus [18F]-fluorodeoxyglucose uptake response in irradiated lung. Int J Radiat Oncol Biol Phys.

[27] Hicks RJ, Manus MP, Mac MJP (2004). Early FDG-PET imaging after radical radiotherapy for non-small-cell lung cancer: inflammatory changes in normal tissues correlate with tumor response and do not confound therapeutic response evaluation. Int J Radiat Oncol Biol Phys.

[28] Rubin P, Casarett GW (1968). Clinical radiation pathology as applied to curative radiotherapy. Cancer.

[29] Coggle JE, Lambert BE, Moores SR (1986). Radiation effects in the lung. Environ Health Perspect.

[30] Gross NJ (1977). Pulmonary effects of radiation therapy. Ann Intern Med.

[31] Gross NJ (1981). The pathogenesis of radiation-induced lung damage. Lung.

[32] Abratt RP, Morgan GW, Silvestri G, Willcox P (2004). Pulmonary complications of radiation therapy. Clin Chest Med.

[33] Zhang G, Meredith TC, Kahne D (2013). On the essentiality of lipopolysaccharide to Gram-negative bacteria. Curr Opin Microbiol.

[34] Castillo R, Pham N, Castillo E (2015). Pre-radiation therapy fluorine 18 fluorodeoxyglucose PET helps identify patients with esophageal cancer at high risk for radiation pneumonitis. Radiology.

[35] Castillo R, Pham N, Ansari S (2014). Pre-radiotherapy FDG PET predicts radiation pneumonitis in lung cancer. Radiat Oncol.

[36] Zhang Y, Yu Y, Yu J (2015). ^18^FDG PET-CT standardized uptake value for the prediction of radiation pneumonitis in patients with lung cancer receiving radiotherapy. Oncol Lett.

[37] McCurdy MR, Castillo R, Martinez J (2012). [18F]-FDG uptake dose-response correlates with radiation pneumonitis in lung cancer patients. Radiother Oncol.

[38] Mac Manus MP, Ding Z, Hogg A (2011). Association between pulmonary uptake of fluorodeoxyglucose detected by positron emission tomography scanning after radiation therapy for non-small-cell lung cancer and radiation pneumonitis. Int J Radiat Oncol Biol Phys.

[39] Alfarouk KO, Verduzco D, Rauch C (2015). Erratum: Glycolysis, tumor metabolism, cancer growth and dissemination. A new pH-based etiopathogenic perspective and therapeutic approach to an old cancer question. Oncoscience.

[40] Batra S, Adekola KU, Rosen ST, Shanmugam M (2013). Cancer metabolism as a therapeutic target. Oncology (Williston Park).

[41] Vander Heiden MG, Cantley LC, Thompson CB (2009). Understanding the Warburg effect: the metabolic requirements of cell proliferation. Science.

[42] Zhang Q, Hu Q, Chu Y (2016). The influence of radiotherapy on AIM2 inflammasome in radiation pneumonitis. Inflammation.

