# Supplementary material for: 18F-Fluorodeoxyglucose positron emission tomography may not visualize radiation pneumonitis
Source: EJNMMI Res. 2019 Dec 19;9:112. doi: 10.1186/s13550-019-0571-0 (PMC6923299; doi:10.1186/s13550-019-0571-0)
Supplement: Supplementary file 1 — Additional file 1: Supplementary Materials. Fig. S1. (A) When performing positioning and radiotherapy, experimental rats were positioned prone in special fixture, three markers were used to determine the location; (B) experimental rats undergoing radiotherapy. Fig. S2. CT scans of irradiated lungs from pre-radiation to 7 weeks after radiotherapy. Fig. S3. Histological changes of irradiated lungs from pre-radiation to 7 weeks after radiotherapy. Fig. S4. Concentrations of inflammatory factors including IL-1, IL-6 and TGF-β in plasma between control rats and irradiated rats. (A) Concentration of IL-1; (B) concentration of TGF-β; (C) concentration of IL-6. **P < 0.01. [file 13550_2019_571_MOESM1_ESM.docx]

**Supplementary Materials**

**Title:** ^18^F-Fluorodeoxyglucose Positron Emission Tomography May Not Visualize Radiation Pneumonitis

Meiying Guo^1,2^, Liang Qi^3^, Yun Zhang^2^, Dongping Shang^2^, Jinming Yu^2^, Jinbo Yue^2*^

1.School of Medicine, Shandong University, Jinan 250012, China

2.Department of Radiation Oncology, Shandong Cancer Hospital and Institute, Shandong First Medical University and Shandong Academy of Medical Sciences, Jinan 250117, China

3.Equipment and material Department, Shandong Cancer Hospital and Institute, Shandong First Medical University and Shandong Academy of Medical Sciences, Jinan, 250117, China

**Journal:** EJNMMI Research

**Correspondence Author:** Jinbo Yue, MD, Department of Radiation Oncology, Shandong Cancer Hospital and Institute, Shandong First Medical University and Shandong Academy of Medical Sciences, No. 440, Ji Yan Road, Jinan 250017, China. Tel.: +86-531-67626442, Fax: +86-531-67626442, E-mail: yuejinbo@hotmail.com.

**Supplementary Method**

**Histology and Immunohistochemistry**

After the micro-PET scan, rats were killed by anesthetizing, and the right lungs were removed, fixed overnight in 10% formalin, and embedded in paraffin. Serial 4-μm tissue sections were prepared for histologic analysis by staining with hematoxylin and eosin. Tissue sections were also prepared for immunohistochemical staining as follows. Briefly, the sections were deparaffinized by three incubations in xylene followed by 5-min incubations in 100%, 95%, and 70% ethanol and rehydrated in water. Endogenous peroxidase activity was blocked with 3% H2O2 for 5 min, then treated with 0.01M citrate buffer (pH 6.0) for 10 min in a microwave oven at 650 W. Slides were incubated with Dual Endogenous Enzyme Block (Agilent, Santa Clara, CA, USA), and then blocked with 1% bovine serum albumin in phosphate-buffered saline/0.1 % Tween-20. The primary antibodies anti-PKM2 (4053, Cell Signaling Technology, Beverly, MA, USA) and rabbit anti-GLUT1 (orb157188, Biorbyt, Cambridge, UK) at dilutions of 1:100 were applied to the slides for 1 h at room temperature, followed by HRP, rabbit/mouse secondary antibody from the EnVision Detection System (Dako). Sections were then counterstained with hematoxylin.

**Supplementary Figure**


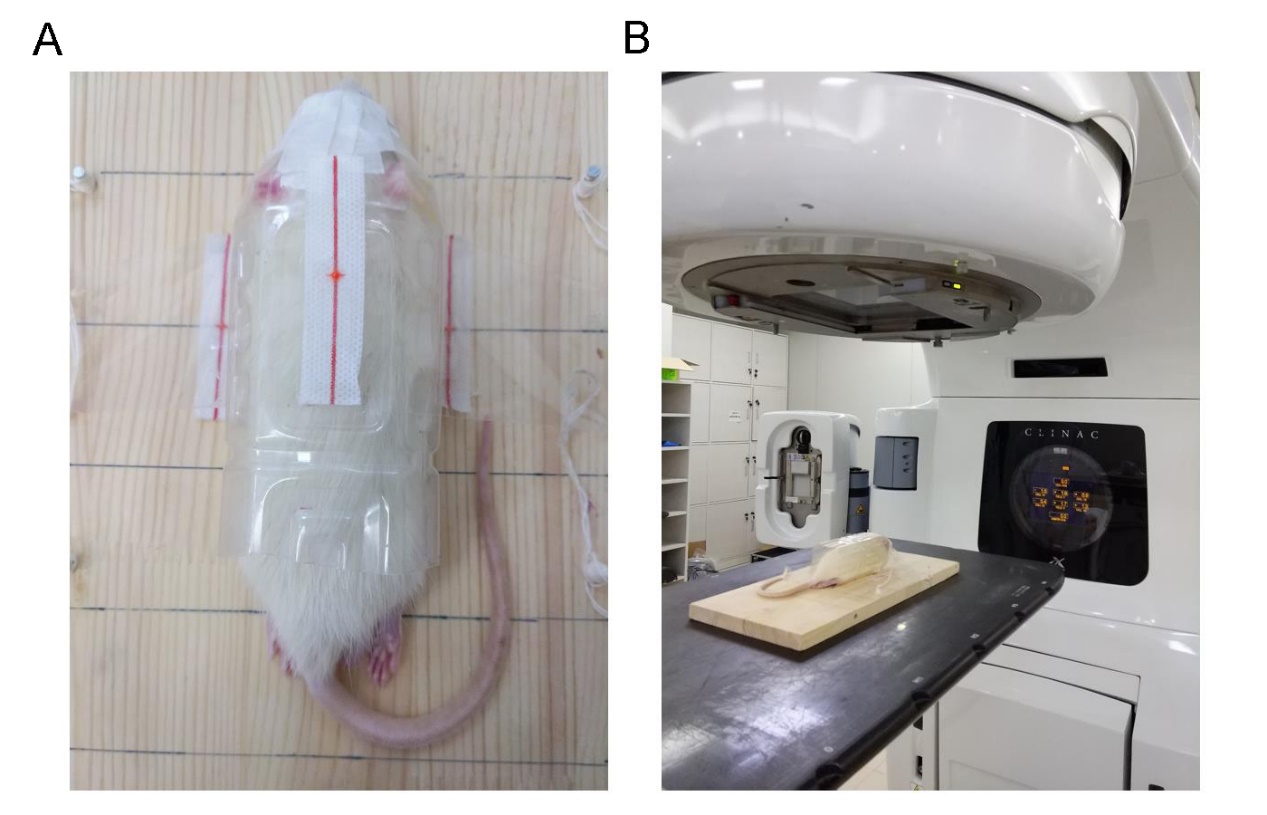


**Suppl. Fig. S1** (A) When performing positioning and radiotherapy, experimental rats were positioned prone in special fixture, three markers were used to determine the location; (B) experimental rats undergoing radiotherapy.


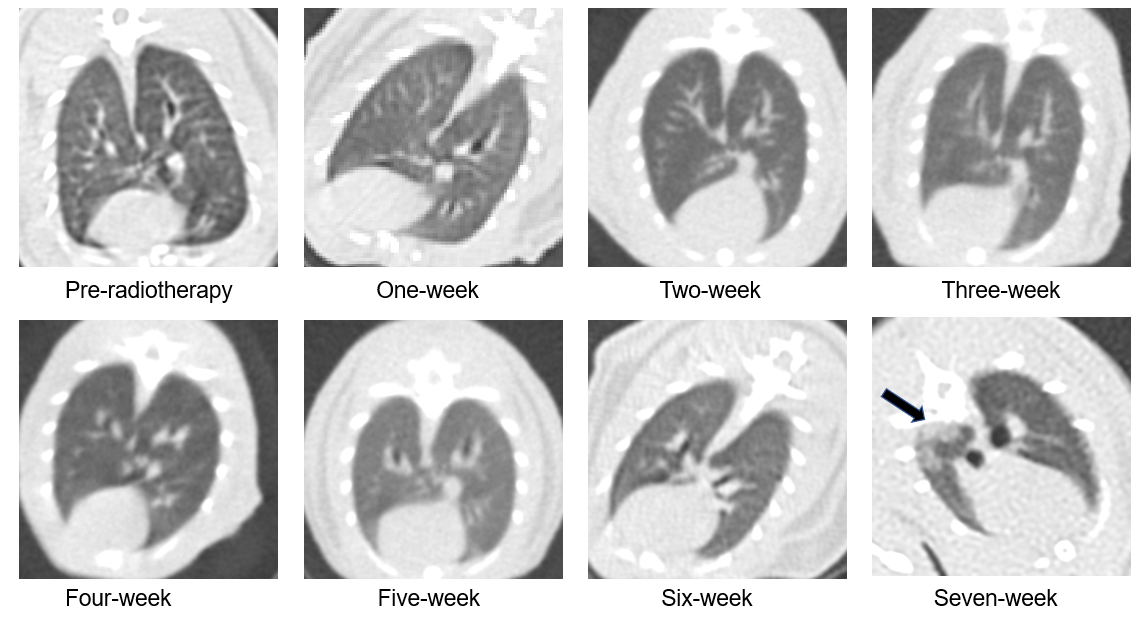


**Suppl. Fig. S2** CT scans of irradiated lungs from pre-radiation to 7 weeks after radiotherapy.


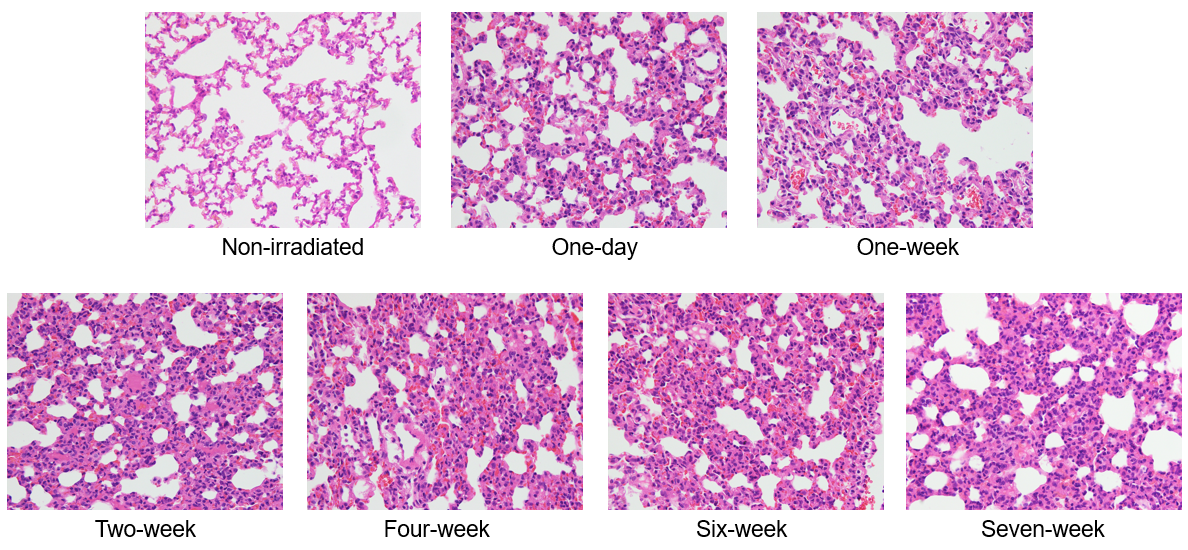


**Suppl. Fig. S3** Histological changes of irradiated lungs from pre-radiation to 7 weeks after radiotherapy.


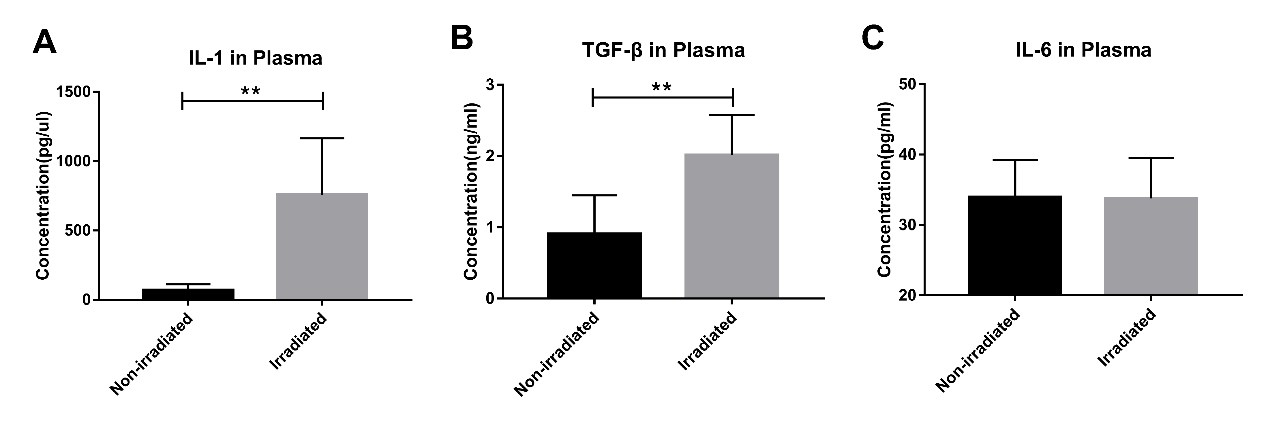


**Suppl. Fig. S4** Concentrations of inflammatory factors including IL-1, IL-6 and TGF-β in plasma between control rats and irradiated rats. (A) Concentration of IL-1; (B) concentration of TGF-β; (C) concentration of IL-6. **P < 0.01.
